# The role of structural bioinformatics resources in the era of integrative structural biology

**DOI:** 10.1107/S0907444913001157

**Published:** 2013-04-19

**Authors:** Aleksandras Gutmanas, Thomas J. Oldfield, Ardan Patwardhan, Sanchayita Sen, Sameer Velankar, Gerard J. Kleywegt

**Affiliations:** a Protein Data Bank in Europe, EMBL–EBI, Wellcome Trust Genome Campus, Hinxton, Cambridge CB10 1SD, England

**Keywords:** structural bioinformatics, integrative structural biology, PDBe

## Abstract

The history and the current state of the PDB and EMDB archives is briefly described, as well as some of the challenges that they face. It seems natural that the role of structural biology archives will change from being a pure repository of historic data into becoming an indispensable resource for the wider biomedical community. As part of this transformation, it will be necessary to validate the biomacromolecular structure data and ensure the highest possible quality for the archive holdings, to combine structural data from different spatial scales into a unified resource and to integrate structural data with functional, genetic and taxonomic data as well as other information available in bioinformatics resources. Some recent developments and plans to address these challenges at PDBe are presented.

## Abstract 2

The integration of structural data on atomistic to cellular scales with genetic, taxonomic and functional information is discussed. The challenges to the PDB and EMDB archives and some pertinent developments at PDBe to address these are discussed.

## 1. Introduction   

Biologists today have an arsenal of three-dimensional imaging tools at their disposal to explore nature at a range of spatial scales and temporal resolutions. Techniques such as X-ray crystallography and nuclear magnetic resonance (NMR) spectroscopy are used to obtain atomic resolution structures of biomacromolecules and their complexes. Electron microscopy (3DEM; Frank, 2009 ▶) and electron tomography (ET; Koning & Koster, 2009 ▶) can be used to image ever larger structures at lower levels of resolution. Light-microscopy techniques can probe even larger structures as well as the real-time dynamics of biological processes (Tomer *et al.*, 2012 ▶).

To fully exploit three-dimensional structural data, we need to annotate its constituent features and understand its context. Three-dimensional ‘snapshots’ of molecular machines provide valuable scientific insight: substructures of functional importance can be identified, for instance the rotary motors in ATP synthase that drive the synthesis of ATP (Okuno *et al.*, 2011 ▶), or the subunits of the ribosome and bound ligands involved in the various stages of RNA-to-protein translation (Rodnina & Wintermeyer, 2011 ▶). Even more revealing is the combination of three-dimensional snapshots into a timeline that shows how a molecular machine carries out its activity. This has been used, for instance, to elucidate the stepwise structural changes that occur in the F_1_ subunit of ATP synthase as it carries out its catalytic activity and, in the case of the ribosome, to study the structural changes and specific ligands that are involved in the initiation, elongation and termination stages of the translation process. Further insight can be gained by ‘zooming out’ in scale and examining these molecular machines in the milieu of the cell. For instance, ET studies of ATP synthase reveal a supramolecular organization of ATP synthase dimers in linear arrays in the mitochondrial cristae, which may be important in ensuring optimal conditions for efficient ATP synthesis (Daum & Kühlbrandt, 2011 ▶; Davies *et al.*, 2011 ▶). In the case of ribosomes, recent ET studies have revealed characteristic packing arrangements along the RNA referred to as polysomes, which are considered to be physiologically important (Brandt *et al.*, 2009 ▶, 2010 ▶).

Integrative structural biology involves the use of multiple structure-determination, modelling and bioinformatics methods to piece together and interpret structural information. Although such studies are invariably carried out to answer a very specific research question, the results obtained can play a valuable role for many years to come, provided that they are properly archived, annotated and linked in a publicly accessible resource. Combining structure-determination methods at different scales enables elucidation of the three-dimensional cellular context of the macromolecular world. Integration from a bioinformatics perspective makes it possible not only to enrich structures with biological information but also to link disparate sources of information and to put the structures in a wider biological context.

In this paper, we briefly describe the history and status of the two prime archives in structural biology, the Protein Data Bank (PDB) and the EM Data Bank (EMDB), some of the challenges that they face in light of the increasing volume, diversity and complexity of data and the crucial need for integration across bioinformatics archives and resources. We also describe a number of important roles that the structural bioinformatics resources play. Finally, we discuss some of the efforts at the Protein Data Bank in Europe (PDBe) to address the challenges associated with the integration of structural and other data and information.

## 2. Structural biology archives   

### 2.1. Protein Data Bank   

Since the early 1970s, atomic coordinates of biomacromolecular structures have been archived in the PDB (Bernstein *et al.*, 1977 ▶; Berman *et al.*, 2000 ▶, 2007 ▶, 2012 ▶). Starting from seven protein crystal structures in 1971, the archive steadily grew over 40 years and now contains over 80 000 structures determined mainly using X-ray crystallo­graphy (87%), NMR spectroscopy (12%) and 3DEM (<1%). For many years, the growth of the archive was essentially exponential (Abad-Zapatero, 2012 ▶). Around the turn of the millennium, the advent of various structural genomics initiatives greatly accelerated the increase in PDB depositions. However, in recent years the growth has been more or less linear (Abad-Zapatero, 2012 ▶). Much of the ‘low-hanging fruit’ has been picked and most structural genomics projects have shifted their focus towards increasingly complex targets. Nonetheless, 9250 new structures were deposited in 2011 alone, more than in the first 25 years of the PDB put together. It is not bold to predict that the archive will continue to grow substantially, not only in terms of the number of entries but also with respect to their size and complexity. The longest observed protein chain in the PDB, found in entry 3vkh describing a motor protein at 3.8 Å resolution (Kon *et al.*, 2012 ▶), contains more than 3000 amino-acid residues (an order of magnitude more than the oldest structures in the archive), and the atomic coordinates of large molecular machines, *e.g.* the different states of a ribosome described in Schmeing *et al.* (2011 ▶), need to be split over several PDB entries owing to limitations of the PDB file format. Table 1 ▶ provides some more highlights and statistics regarding the contents of the PDB and EMDB archives.

For the first two decades of its existence, depositions to the PDB were either voluntary or mandated by a few journals, with IUCr publications taking the lead and others following suit in requiring that all reported model coordinates be deposited in the PDB. However, in the 1980s a number of high-profile cases demonstrated that not all published structures were reliable (Brändén & Jones, 1990 ▶). It became clear that coordinates alone were not sufficient to verify the correctness of the interpretation of the underlying experimental data and hence the validity of any biological claims. The case for the deposition of models and experimental data has been made many times and the arguments in favour (Kleywegt *et al.*, 2004 ▶) include (i) that experimental data may contain features amenable for alternative interpretations to the deposited model; (ii) the facilitation of assessing specific claims; (iii) the possibility of a more thorough validation and distinction between genuine outliers and errors in interpreting the data; (iv) the facilitation of scientific progress, including follow-up studies on a given molecule and methods development; (v) the archival and retrieval of the data, including by the group who contributed the data earlier; and (vi) that the format uniformity assured by the database can facilitate large-scale processing and data mining. Once again, the IUCr and its publications took the lead in mandating the deposition of experimental structure-factor amplitudes or intensities, but it took a further number of embarrassing cases including both serious errors in protein structures (Chang *et al.*, 2006 ▶) and suspected or demonstrated cases of scientific fraud before the wider X-ray community was willing to follow suit. Since 2008, the deposition of experimental X-ray and NMR data is mandatory when models are deposited in the PDB, and most journals follow the IUCr guidelines (Commission on Bio­logical Macromolecules, 2000 ▶).

**Figure 1:**
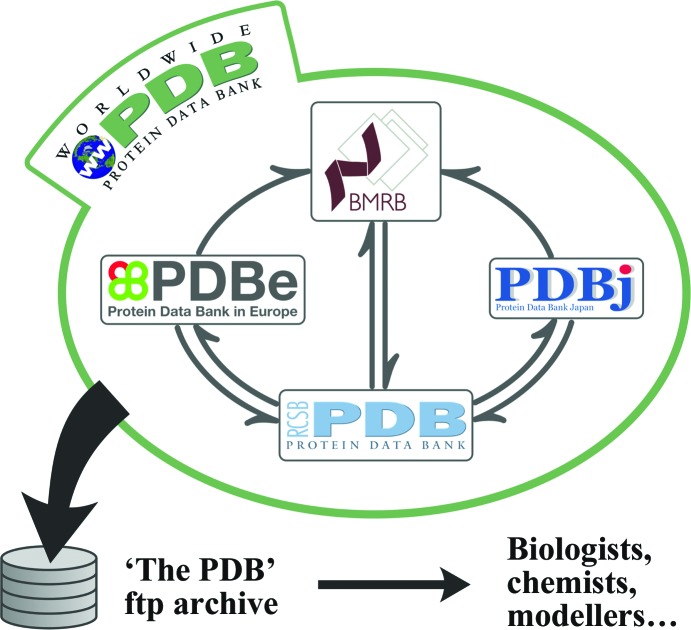
The wwPDB partner sites collaborate on annotating, archiving, managing and distributing the PDB data, ensuring a single global, freely accessible archive.

The management of the PDB archive has changed dramatically in the past 10–15 years in response to shifting demands stemming from the enormous growth and complexity of the structural biology field as a whole. For the first two decades, the PDB was housed at and maintained by Brookhaven National Laboratory (BNL; Bernstein *et al.*, 1977 ▶), but in 1998 the management of the PDB was taken over by the Research Collaboratory for Structural Bioinformatics (RCSB PDB; Berman *et al.*, 2000 ▶) at Rutgers University and the University of California at San Diego (initially, the National Institute of Standards and Technology was also a partner). In 2003, the way in which the PDB archive was managed was transformed by the establishment of the Worldwide Protein Data Bank organization (wwPDB; http://wwpdb.org; Berman *et al.*, 2003 ▶). Its founding partners were the RCSB PDB (Berman *et al.*, 2000 ▶), the Protein Data Bank Japan (PDBj; Kinjo *et al.*, 2012 ▶) and the Protein Data Bank in Europe (PDBe; Velankar *et al.*, 2011 ▶, 2012 ▶). In 2006, they were joined by the Biological Magnetic Resonance Bank (BMRB; Ulrich *et al.*, 2008 ▶; Markley *et al.*, 2008 ▶). The four wwPDB partner sites now jointly manage essentially all aspects of the archive (see Table 2 ▶). Major areas of collaboration include managing the policies and issues related to the deposition and annotation of biomacromolecular structures, data formats, standards and data validation. In addition, the wwPDB partners maintain reference data such as the descriptions of small molecules and nonstandard residues that are found in the PDB. Fig. 1 ▶ shows the interactions between the partners and emphasizes the difference between the archive (PDB, an ftp tree of flat files) and the organization that manages it (wwPDB, made up of four equal and independent partner organizations).

There is one area in which the wwPDB partners do not collaborate but engage in friendly competition, namely the dissemination of the archive data through means other than the ftp archive of flat files. Each partner has typically organized the PDB data in a professionally run relational database management system and developed their own websites, which allow efficient searches of the archive, expose individual PDB entries to users (often with value-added information specific to that partner) and provide advanced services utilizing the PDB data.

The various roles that structural bioinformatics resources and organizations such as PDBe, wwPDB and EMDataBank (Lawson *et al.*, 2011 ▶) play are summarized in Table 3 ▶ and discussed in §[Sec sec3]3.

### 2.2. Electron Microscopy Data Bank (EMDB)   

In 2002, the EMDB (Tagari *et al.*, 2002 ▶) was founded at the EBI specifically to archive the non-atomistic structures (*i.e.* volume maps, masks and tomograms) determined by a variety of 3DEM methods, including single-particle methodology, ET and electron crystallography. Today, the EMDB contains over 1300 released entries and is expected to grow 5–10-fold by 2020. The lion’s share of entries are the result of single-particle studies, while tomography accounts for only 6%. Since 2007, the EMDB has been managed jointly under the aegis of the EMDataBank organization (http://emdatabank.org; Lawson *et al.*, 2011 ▶) by three partners: PDBe, RCSB PDB and the National Center for Macromolecular Imaging (NCMI) at Baylor College of Medicine. The relationship between EMDB (the archive of EM volume data) and EMDataBank (the organization that manages it) is analogous to the relationship between the PDB archive and the wwPDB organization.

Whereas deposition of experimental data in the PDB is mandatory for X-ray and NMR structures, not all journals and funding agencies apply the same demand to 3DEM studies. Analysis of deposition behaviour for the 2011 volumes of a number of relevant journals shows that for single-particle EM studies almost 50% were deposited in the EMDB, but only 30% of published tomographic reconstructions ended up in the EMDB archive (see Table 4 ▶). For the future of (integrative) structural biology it is vital that the attitude of the community to deposition is improved or the data are likely to be lost forever. The EMDataBank has consulted extensively with the EM community to understand the reasons for the paucity of depositions and to solicit ideas on how to improve these numbers. PDBe and Open Microscopy Environment (OME; http://www.openmicroscopy.org; Allan *et al.*, 2012 ▶) organized a workshop on Data-Management Challenges in 3D Electron Microscopy (DMCEM; Patwardhan *et al.*, 2012 ▶) to engage with leaders in the EM field, resulting in substantial input to and endorsements of the important role that the EMDataBank plays. The meeting also encouraged the EMDataBank to engage more with the tomography community to clarify deposition policies and to encourage deposition. The EMDataBank therefore organized a special discussion session at the 2012 3DEM Gordon Research Conference that resulted in a policy text that was later circulated to the wider EM community through the 3DEM mailing list and was met there with unanimous approval. Moreover, input has been solicited from members of the 3DEM community on how the EMDB data model can best accommodate the particularities of tomographic experiments and the deposition of segmentations. Looking ahead, the archiving needs and opportunities for emerging high-resolution cellular imaging techniques need to be considered, such as three-dimensional scanning electron microscopy (3DSEM), soft X-ray tomography (SXT) and super-resolution light microscopy, as well as techniques for correlating imaging data on the cellular and macromolecular scales. The development of archival resources for these techniques will be vital for providing a three-dimensional cellular context to the macromolecular world and thereby enabling true integrative structural biology.

### 2.3. Challenges for the data archives   

The increasing volume, diversity and complexity of bio­logical data has challenged structural biology archives to efficiently manage these data and to make them accessible to an increasingly large and diverse community of users (who are not necessarily all experts in structural biology). In the case of the PDB, the increasing size and complexity of the biomacromolecules studied by the research community, the recent advances in different experimental methods and the emergence of hybrid techniques to obtain structural insights into biologically relevant molecules, complexes and molecular machines all present major challenges for the management and presentation of the valuable data contained in the archive. To address some of these challenges, the wwPDB partners are developing a common software system that will allow deposition, validation and annotation of complex and diverse macromolecular structures along with the underlying experimental data using a single interface (see §[Sec sec3.1]3.1). The efforts to validate experimental and associated metadata at the time of deposition will not only improve the quality of the archives (PDB, EMDB and BMRB) but will also help in delivering structural data to users with no or limited structural biology background (Velankar & Kleywegt, 2011 ▶). These efforts also facilitate the integration of structural data with other biological data by identifying, for instance, the best currently available structure of a given protein. Historically, the PDB has been an archive of structural data as described in the associated publications and it has primarily served X-ray crystallographers and other structural biologists. In the long term, the role of the PDB may well need to change from a provider-centric archive to a user-centric biomedical resource (Velankar & Kleywegt, 2011 ▶). Such a shift of focus requires not only improved data capture (which is one of the main goals of the new wwPDB deposition and annotation system) but also improved ways to deliver biomacromolecular data to the wider biomedical community (and related fields, such as agriculture). Improved integration of structural data with other biological data resources will stimulate the development of new ways to deliver biomacromolecular structure data. In addition, rapid advances in model-building, refinement and validation methodology in the last decade have resulted in the development of automated protocols such as *PDB_REDO* (Joosten *et al.*, 2012 ▶) that produce models for the vast majority of crystal structures in the PDB that are superior to the models that were originally published and deposited. Such developments make it possible to offer state-of-the-art structural models to the community rather than (or at least in addition to) the historic data archived in the PDB.

### 2.4. The Protein Data Bank in Europe (PDBe)   

PDBe is one of the core resources of the European Bio­informatics Institute (EMBL–EBI; Brooksbank *et al.*, 2010 ▶). The mission of the PDBe is *bringing structure to biology*, *i.e.* to enable scientists with limited structural biology expertise to use biomacromolecular structure data that are relevant to their research in a multitude of ways, while also providing advanced tools for expert structural biologists. An important part of fulfilling this mission is to provide web-based tools, services and resources, many of which have been discussed previously (Velankar & Kleywegt, 2011 ▶; Velankar *et al.*, 2011 ▶, 2012 ▶).

The primary function of the PDBe website is to allow efficient searches of PDB and EMDB data and to expose individual entries from both archives to the end users in an intuitive way. Lists of search results and summary pages for each entry include the PDBprints widget (Velankar *et al.*, 2011 ▶), which gives pictorial representations of the content and origin of the entry. The summary pages also feature PDBportfolio (Velankar *et al.*, 2012 ▶), a set of snapshots that highlight interesting aspects of the three-dimensional structure, such as the ligand-binding environment, domains and quaternary structure. PDBe has enhanced the capabilities of the OpenAstexViewer (Hartshorn, 2002 ▶; Oldfield, 2004 ▶) and now uses it extensively for interactive three-dimensional visualization of structures, including EM and NMR data, and for educational purposes (Quips tutorials; Velankar *et al.*, 2012 ▶).

Besides basic search functionality, PDBe offers a number of tools for advanced analysis of individual entries or the entire PDB archive. *PDBePISA* (Krissinel, 2011 ▶; Krissinel & Henrick, 2007 ▶) deduces the most probable quaternary structure for any crystal structure in the PDB based on free-energy analysis of all possible interacting surfaces, taking symmetry into account. *PDBeFold* (Krissinel & Henrick, 2004 ▶) is a server for rapid structure-similarity searches which allows both pairwise and multiple structure alignment and which can be used to identify proteins that show (partial) three-dimensional similarity to a structure of interest. *PDBeMotif* (Golovin & Henrick, 2008 ▶) is an advanced service that combines information regarding chemistry, active sites, protein sequence and structure in a single tool that allows a large variety of complex searches across the PDB down to the level of individual atoms and their interactions. Unfortunately, there is a somewhat steep learning curve for *PDBeMotif*, which is why a simple front-end has been developed. This service, *PDBeXpress* (Velankar *et al.*, 2012 ▶), provides a very simple interface to carry out a number of popular queries that are well defined but are limited in scope. Examples include searching for ligands that bind to a particular set of amino acids or, inversely, finding out which residue types are most commonly found to interact with a particular ligand.

PDBe provides a wide range of services based on EMDB through its EM portal, including advanced search (EMsearch), statistics (EMstats) and visualization. The OpenAstexViewer-based EM volume viewer makes it possible to explore large EMDB maps interactively in a browser window. Visual analysis pages provide map projections, map–model overlays, density-distribution charts and atom-inclusion plots, thus serving as a crude validation tool of EMDB maps and associated PDB models. More recently, we have introduced a tomogram slice viewer that allows scrolling and zooming through tomographic reconstructions without any need for special software or expertise.

For NMR entries in the PDB, we provide value-added services such as cluster analysis of the deposited ensemble and identification of rigid domains in the structure (*OLDERADO*; Kelley & Sutcliffe, 1997 ▶). This service also presents the most representative model for the NMR ensemble and for each cluster. Analysis of chemical shift data includes the correction of systematic errors and the identification of unusual chemical shift values based on the amino-acid type and solvent accessibility for each atom (*VASCO*; Vranken & Rieping, 2009 ▶). The output of both the *OLDERADO* and the *VASCO* services as well as analyses of deposited experimental constraints and NRG-CING (Doreleijers *et al.*, 2012 ▶) validation reports can be studied using an interactive three-dimensional viewer (*Vivaldi*; Velankar *et al.*, 2012 ▶; Hendrickx *et al.*, 2013 ▶).

## 3. Roles of structural bioinformatics resources   

Structural bioinformatics resources, both individually and as international collaborations such as wwPDB and EMDataBank, play a multitude of roles in the field of structural biology besides managing the structural biology archives. Some of these roles are entrusted to them because they are generally regarded as independent and not in any direct competition with structural biology laboratories; other roles fit naturally with their function as archive managers. Table 3 ▶ lists a number of areas in which organizations such as PDBe (and its counterparts in other continents), wwPDB and EMDataBank have important roles to play, as well as some concrete examples of these.

### 3.1. The wwPDB common deposition and annotation tool   

The wwPDB partners are jointly developing a common deposition and annotation (D&A) tool that will be used at all wwPDB sites to annotate structural data produced by any combination of experimental techniques (at present X-ray diffraction, neutron diffraction, NMR spectroscopy, EM, ET and electron crystallography). This is a shift from the current practice of having four different software systems (*ADIT* at RCSB and PDBj, *ADIT-NMR* at BMRB and PDBj-BMRB, *AutoDep* at PDBe and *EMDep* at PDBe and RCSB) to process depositions to the PDB, BMRB and EMDB archives. The common tool also enables load-balancing between the processing sites. An interactive and informative deposition interface will streamline deposition of data to the archives. Compared with the current systems, the new software will offer enhanced functionality, and future extensions for handling new experimental techniques will be relatively easy to implement. The depositors will access their deposition sessions *via* password-protected logins and communicate with wwPDB annotation staff through the deposition interface, thus eliminating the current practice of emailing replacement coordinates for entries that are still being annotated. Validation will become an integral part of the deposition and annotation process (see §[Sec sec3.2]3.2). Once a structure has been deposited it will go through the process of annotation followed by approval and public release. The weekly PDB release takes place every Wednesday at 00:00 UTC. The released PDB entries are made publicly available *via* ftp by all the wwPDB partner sites simultaneously.

### 3.2. Validation of deposited structures   

Despite the availability of easy-to-use validation tools such as *PROCHECK* (Laskowski *et al.*, 1993 ▶), *WHATCHECK* (Hooft *et al.*, 1996 ▶), *OOPS* (Kleywegt & Jones, 1996 ▶) and *MolProbity* (Chen *et al.*, 2010 ▶) since the early 1990s, cases of serious errors in published structures continue to come to light to this day. To improve the validation of structures ‘at the gate’, the wwPDB partners have established validation task forces (VTFs) for X-ray crystallography and NMR spectroscopy (Berman *et al.*, 2010 ▶), while EMDataBank has convened a cryo-Electron Microscopy VTF (Henderson *et al.*, 2012 ▶). The recommendations of these expert committees reflect broad community consensus on the core statistics for each experimental method that measure the quality of the model, the experimental data and the fit of the model to the data. The recommendations of the X-ray VTF (Read *et al.*, 2011 ▶) are being implemented in a dedicated validation pipeline (Gore *et al.*, 2012 ▶), which will be integrated with the new wwPDB D&A system and will also be made available as an anonymous server. Similar pipelines will be developed for NMR and EM depositions as and when the expert recommendations become available. The pipelines will include tools for validation of the experimental data and macromolecular models deposited in the PDB such as *MolProbity* (Chen *et al.*, 2010 ▶), *PHENIX* (Adams *et al.*, 2010 ▶), *EDS* (Kleywegt *et al.*, 2004 ▶) and *WHATCHECK* (Hooft *et al.*, 1996 ▶). Moreover, the quality of the small-molecule data in the archive will be improved by the use of the Cambridge Crystallographic Data Centre (CCDC; http://www.ccdc.cam.ac.uk/) program *Mogul* (Bruno *et al.*, 2004 ▶) for ligand-geometry validation. The pipelines will produce a validation report (PDF file) that can be made available to editors and referees of manuscripts and an XML file that contains all the details.

### 3.3. Handling new experimental methods   

In the past, structural biology experts have usually focused on the use of one particular experimental technique, such as X-ray crystallography or 3DEM. Increasingly, however, biologists want to ask questions that require structural information without being limited to one or two techniques. Consequently, new techniques and new applications of existing techniques emerge all the time and combinations of techniques (so-called hybrid methods) are used to produce low-resolution models of complexes and large molecular machines (Alber *et al.*, 2007*a* ▶,*b* ▶). Examples of such techniques include small-angle neutron and X-ray scattering (SANS/SAXS), fluorescence resonance energy transfer (FRET), electron paramagnetic resonance (EPR), circular-dichroism spectroscopy (CD) and soft X-ray tomography. Not all of these techniques produce atomistic models in themselves: often they contribute supportive or complementary structural data. It is important to establish guidelines about the kinds of models and experimental data that should and should not be archived in the PDB. The wwPDB partners have convened a task force involving experts from the small-angle scattering (SAS) field, which had its first meeting in the summer of 2012 and will provide recommendations about the archiving needs and requirements for SAS-based models and about mandatory data and validation criteria for structures solved using SAS as a sole experimental technique or in combination with other methods, *e.g.* NMR. A similar wwPDB task force for hybrid methods will be convened in 2014.

### 3.4. File-format specifications   

The PDB format, while being easily human-readable, is more than 40 years old and does not support the needs of today’s science for data representation, *e.g.* it limits the number of macromolecular chains to 36 and the number of atoms to 99 999, and it does not support description of chirality and bond orders or data from nondiffraction techniques. It is thus unable to properly represent large structures (*e.g.* a ribosome) or to properly represent the chemistry of ligands. In September 2011, wwPDB organized a meeting with several key software-development teams in the X-ray field to agree on a future-proof replacement for the PDB format and to discuss supporting software requirements. The meeting participants agreed to adopt the existing PDBx/mmCIF format, which does not suffer from the above restrictions and for which an extensive software base already exists, rather than to develop and introduce a completely new format. A working group has been set up to meet the requirements for adoption of the PDBx/mmCIF format in major macromolecular crystallo­graphic software tools and during deposition to the PDB. The future transition from PDB format to PDBx/mmCIF will be carried out in consultation with all major stakeholders.

### 3.5. Data models and ontologies   

The mmCIF dictionary (Bourne *et al.*, 1997 ▶) was initially developed as an extension to the core CIF dictionary (Hall *et al.*, 1991 ▶) with a view to accommodating complex relationships between data items describing macromolecular structures. In light of the need for consistent representation and archiving of associated experimental data, mmCIF is therefore used as the data model for the PDB. The wwPDB partners have been involved in the extension of the mmCIF dictionary to represent all of the data managed and distributed by them, including data items specific to NMR and EM experiments, protein-production protocols *etc.* The PDB Exchange dictionary (PDBx) and the PDB archival data files are also available in an XML format known as PDBML (Westbrook *et al.*, 2005 ▶).

The current archival format for NMR experimental data at BMRB (NMR-STAR; http://bmrb.wisc.edu/dictionary) is very comprehensive; however, it is often impractical to use as an exchange format between different NMR software. To address this problem, the Collaborative Computing Project for the NMR community (CCPN; Fogh *et al.*, 2002 ▶) has developed a versatile data model (Vranken *et al.*, 2005 ▶). The CCPN *FormatConverter* software delivers the ability to exchange data and the Entry Completion Interface (ECI; Penkett *et al.*, 2010 ▶) helps to prepare CCPN projects for deposition at PDBe and exports an NMR-STAR file for submission to BMRB.

Despite the fact that 87% of the structures in the PDB have been determined by X-ray crystallography, the crystallization of biological samples largely remains a ‘trial-and-error’ method and the results of all of the crystallization trials are rarely captured during deposition or in publication. To address this problem, a crystallization data-exchange workshop (XDX) was organized by several international large-scale crystallization-screening laboratories (Newman *et al.*, 2012 ▶). This meeting resulted in a proposal for an ontology to describe information relating to the crystallization experiment (*e.g.* the chemical and physical conditions, the methods used and the outcome of the experiment). Formalizing the data in this manner will facilitate data mining on a potentially large body of data which would otherwise remain inaccessible to most users.

### 3.6. Community interactions and challenges   

In order to provide optimal services to the structural biology and various other user communities, the archival resources tend to have many and close interactions with the wider scientific community (see Table 3 ▶ for examples). The issues relating to the management of the PDB and EMDB archives, policies, formats, interactions with journals and the like naturally fall under the aegis of the wwPDB and EMDataBank organizations and their advisory committees. If and when necessary, these organizations also call upon community experts to advise on matters of policy. The various validation task forces are prime examples of this, as is the format workshop mentioned earlier. In 2011, the PDBe and OME teams arranged a consultative workshop with key members of the EM community on Data-Management Challenges in 3D Electron Microscopy (DMCEM) to discuss a range of issues including data formats, raw data archiving and EM validation, and made a number of recommendations in these areas (Patwardhan *et al.*, 2012 ▶).

In the UK, PDBe is collaborating actively with CCPN (Fogh *et al.*, 2002 ▶), has long-standing interactions with CCP4 (Winn *et al.*, 2011 ▶) and was involved in the founding of CCP-EM (http://www.ccpem.ac.uk/). From a European perspective, PDBe functions as the interface between two key biomedical infrastructure projects, Elixir (http://www.elixir-europe.org) for bioinformatics and Instruct (http://www.structuralbiology.eu) for structural biology.

Structural bioinformatics resources are also often involved in community-challenge projects. For example, CAPRI (Critical Assessment of PRediction of Interactions; http://pdbe.org/capri; Janin & Wodak, 2007 ▶) is hosted by PDBe, structure depositors at RCSB and PDBj can opt to submit their structures to CASP (Critical Assessment of Protein Structure Prediction; Moult *et al.*, 2011 ▶), the EM Modelling Challenge (Ludtke *et al.*, 2012 ▶) was organized by the three EMDataBank partners, and PDBe was involved in the Critical Assessment of Structure Determination by NMR challenge (CASD-NMR; Rosato *et al.*, 2012 ▶).

## 4. Integrative structural biology   

**Figure 2:**
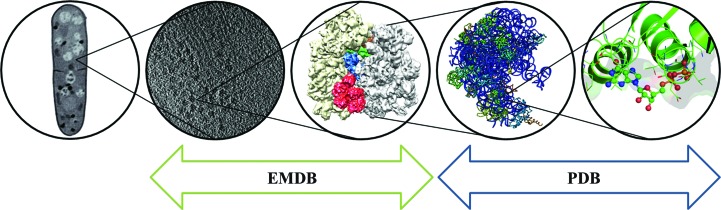
The scales of structural biology and their relationship to the currently available archives (EMDB and PDB): from the atomic details and interactions in a binding site to the cellular context of molecular machines, illustrated using the ribosome as an example. From left to right: soft X-ray tomogram of a fission yeast cell (adapted from Larabell & Nugent, 2010 ▶ with permission of Elsevier), electron tomogram of ribosomes in the cytosol (EMDB entry EMD-5227; Brandt *et al.*, 2010 ▶), cryo-EM reconstruction of the 80S ribosome from yeast (EMDB entry EMD-2008; Becker *et al.*, 2012 ▶), crystal structure of the 50S ribosomal subunit (PDB entry 3uzk; Demeshkina *et al.*, 2012 ▶) and crystal structure revealing how tmRNA and the small protein SmpB enable the kirromycin-stalled 70S ribosome to proceed with translation (PDB entries 4abr and 4abs; Neubauer *et al.*, 2012 ▶).

The structural biology and bioinformatics fields face dual challenges as regards integration. Firstly, it now becomes possible to integrate three-dimensional structural data from a continuum of spatial scales from atomic to cellular obtained using a large arsenal of experimental techniques (Fig. 2 ▶). Secondly, for the structural data to be useful beyond the scope and lifetime of the project in which they were collected, it is vital to link together disparate bioinformatics resources and to provide useful, usable, accurate and up-to-date functional, genetic and taxonomic annotation. This section addresses both challenges in more detail.

### 4.1. Integration from a structural perspective   

Many structural studies involve the use of more than one structural technique. For instance, X-ray crystallography and NMR may be employed to obtain structures of individual proteins, with single-particle EM being used to examine the structure of the entire complex and ET being used to examine their organization in the context of the cell (Fig. 2 ▶). An illustrative example is the ribosome. NMR and X-ray crystallography have been used to obtain atomic resolution structures of individual ribosomal proteins (Stoldt *et al.*, 1998 ▶; Nikulin *et al.*, 2003 ▶), single-particle EM has been used to obtain maps of ribosomal complexes in various functional states, a few ribosomal complexes have been solved to atomic resolution by X-­ray crystallography (Wimberly *et al.*, 2000 ▶; Selmer *et al.*, 2006 ▶; Ban *et al.*, 2000 ▶) and now electron tom­ography is being used to study the organization of ribosomes in the cytoplasm along nascent mRNA (Brandt *et al.*, 2009 ▶, 2010 ▶). The new wwPDB D&A system has been designed with the assumption that hybrid structures (based on experimental data obtained by more than one technique) will be the rule rather than the exception in the future. From the perspective of presenting structural data to the users, it is important that the relationships between structures obtained using different techniques are either recorded or can be mined from the data itself. Determining such relationships is relatively straight­forward for high-resolution structures that can be interpreted in terms of atomic coordinates. However, although the resolution achievable by EM techniques has improved dramatically over the past ten years, only a handful of structures have been determined for which the map can be directly interpreted in terms of an atomic model. More typically, the best that can be done is to fit known atomic models into the map as rigid bodies. If this is not possible, the map can be segmented into regions and the regions annotated with pertinent database identifiers that can help to link the region with other structures. For instance, associating a region of density with a UniProt identifier will help link it to any structures with the same UniProt identifier, even if such structures are determined ten years after the EM map was deposited. The support for segmentation information in the EMDB archive is currently very poor. Individual segments can be uploaded as separate files but without any consistent biological annotation, making it virtually impossible to link them to other resources. One outcome of the DMCEM workshop (see §[Sec sec3.6]3.6) was an agreement that the EMDataBank would draft a new segmentation file format to support bio­logical annotation of the segmented regions in a way that makes it easy to link them to other biological resources, including the structural ones.

**Figure 3:**
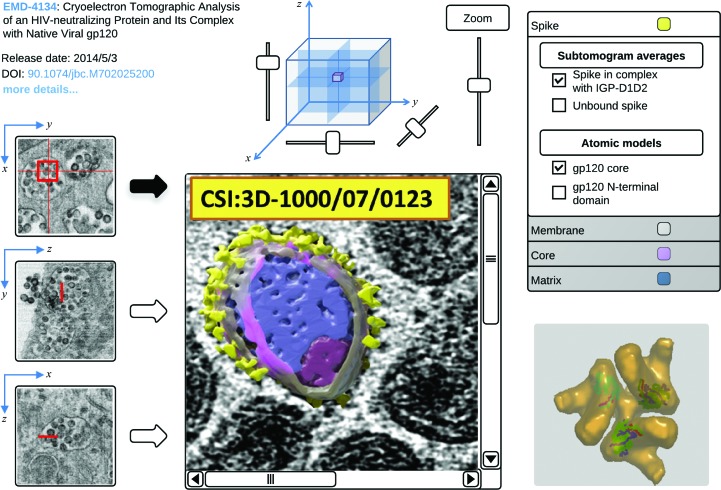
Mock-up of what a volume browser for three-dimensional cellular imaging data could look like, using HIV/SIV as an example. The three leftmost images are derived from a 3DSEM reconstruction and show HIV virion reservoirs in infected macrophages (from Bennett *et al.*, 2009 ▶; adapted under the terms of the Creative Commons Public Domain declaration). These three orthogonal cross-sections and the cube help users to orient themselves in the data. The central panel shows a slice from a cryo-electron tomographic reconstruction in which the features of individual SIV virus particles can be identified (from Bennett *et al.*, 2007 ▶; adapted under the terms for noncommercial use; http://www.jbc.org/site/misc/Copyright_Permission.xhtml). Here, biologically meaningful segmentations have been overlaid on the tomogram and the corresponding annotations are shown in the top right panel. The bottom right panel shows a three-dimensional rendering of data from EMDB (gold-coloured density from a sub-tomogram average of a HIV viral spike; EMDB entry 5018; Liu *et al.*, 2008 ▶) and PDB (the fitted atomic model inside it; structure of a HIV-1 gp120 trimer; PDB entry 3dno; Liu *et al.*, 2008 ▶).

At present, information about the cellular context of the macromolecules in the PDB and EMDB is available at the level of metadata. For instance, the SIFTS resource (Velankar *et al.*, 2005 ▶, 2013 ▶; see also §[Sec sec4.2]4.2) provides for almost every protein (fragment) in the PDB the appropriate GO terms that describe its cellular localization. The ability to capture bio­logically annotated segmentation information will make it possible to exploit the tomography data in the EMDB archive to provide a three-dimensional structural perspective on the cellular context of biomacromolecules and their complexes. To provide this perspective to non-expert users, PDBe plans to develop a tomogram browser which will be able to overlay segmentation data and provide links to other resources (see Fig. 3 ▶ for a mock-up). However, even in the absence of segmentations, map data can be exploited to find relationships between structures, namely by matching three-dimensional shapes to a database of known structures, segmentations and maps. As part of a collaboration between Instruct and Elixir, a database of EMDB and PDB shape data will be created and shape-matching will be developed to allow mining of the structural archives based on non-atomistic data.

Besides ET, a number of imaging techniques have emerged in recent years (such as X-ray tomography, automated serial-section EM techniques and correlative fluorescence microscopy/EM) that promise to provide an unparalleled three-dimensional structural perspective on the cellular context of biomacromolecules and their complexes. PDBe will strive to exploit the opportunities presented by these developments and to develop archiving and visualization tools to link between imaging scales.

### 4.2. Integration from a bioinformatics perspective   

For over a decade, major efforts in bioinformatics have focused on integrating diverse and complex biological data to provide the research community with a platform to understand complex biological phenomena (Chicurel, 2002 ▶). Various technologies and approaches such as data warehousing, federated database systems, service-oriented architecture and, more recently, semantic web technologies have been developed and used to address the issue of integration of distributed heterogeneous biological data resources. The aim is to make it possible for researchers to access a wide range of biological data to help understand biological phenomena. EMBL–EBI is home to many biological data resources (Brooksbank *et al.*, 2010 ▶), which puts PDBe in a favourable position to integrate the biomacromolecular structure data in PDB and EMDB with other biological resources to enhance their biological annotations.

For over a decade, PDBe and UniProt (UniProt Consortium, 2012 ▶) have worked together to integrate information from protein sequences and structures, resulting in a data resource called SIFTS (Structure Integration with Function, Taxonomy and Sequences; Velankar *et al.*, 2005 ▶, 2013 ▶). This resource provides up-to-date residue-level annotation of protein structures in the PDB with data available from UniProt, InterPro (Hunter *et al.*, 2009 ▶), Pfam (Punta *et al.*, 2012 ▶), GO (Ashburner *et al.*, 2000 ▶), CATH (Cuff *et al.*, 2011 ▶) and SCOP (Andreeva *et al.*, 2008 ▶). The data are distributed in XML format and are used by many research and service teams around the world such as the RCSB PDB, Pfam, CATH and DAS server providers (http://www.dasregistry.org). Future integration of information from resources such as Ensembl, which contains data on single-nucleotide polymorphisms (SNPs; Flicek *et al.*, 2012 ▶), IntAct, which provides macromolecular interaction data (Kerrien *et al.*, 2012 ▶), and Reactome, which describes biological pathways (Croft *et al.*, 2011 ▶; Matthews *et al.*, 2009 ▶), will make structural data available in genomic, proteomic and biological pathway contexts.

SIFTS has made it possible to develop intelligent query and visualization mechanisms to present structures in biological contexts that make the structural data more relevant and accessible for the wider biomedical field. One such development, the PDB archive browser PDBeXplore (Velankar *et al.*, 2011 ▶), organizes and presents structural data based on well known biological classifications, with additional analysis of the selected structures. At present, there are six browser modules based on the following classification systems:

(i) The Enzyme Classification (EC) system as included in the Intenz database (Fleischmann *et al.*, 2004 ▶; http://pdbe.org/ec).
(ii) The sequence-based protein-family classification system Pfam (Punta *et al.*, 2012 ▶; http://pdbe.org/pfam).
(iii) The fold-based protein-family classification system CATH (Cuff *et al.*, 2011 ▶; http://pdbe.org/cath).
(iv) Gene Ontology (GO) assignments of molecular function, cellular component and biological processes (The Gene Ontology Consortium, 2000 ▶; http://pdbe.org/go).
(v) Taxonomic data from the NCBI taxonomy database (Sayers *et al.*, 2012 ▶; http://pdbe.org/taxonomy).
(vi) Analysis of all PDB entries that contain a given chemical compound (http://pdbe.org/compounds).

The browser not only shows the relevant PDB entries but also the distribution of probable quaternary structures, bound ligands, sequence-family data, taxonomy and fold classifications. The information can be downloaded for further analysis. In addition, functionality to browse PDB entries containing proteins with similar sequences (http://pdbe.org/fasta) is provided. Future extensions to SIFTS will be reflected in concomitant enhancements of the PDBeXplore browser.

## Figures and Tables

**(a):** PDB holdings on 12/12/2012.

	Total	X-ray	NMR	EM
No. of structures	86785	76355†	9727†	520
Highest resolution X-ray structure		0.48 (3nir)		
Length of longest observed polymer chains				
Protein		3040 (3vkh)	828 (2vda)	1630 (3iyv and 1xi4)
RNA		3206 (3o5h and 3o58)	111 (2lkr)	3352 (3izf)
DNA		347 (1zbb)	42 (2f1q)	4896 (2ymf)
PDB entry with the highest number of				
Polymer residues	89160 (split over 4fy1, 4fy2, 4fy3, 4fy4, 4fy5, 4fy6, 4fy7, 4fy8, 4fy9, 4fya)			
Macromolecular atoms	717805 (split over 1voq, 1vor, 1vos, 1vou, 1vov, 1vow, 1vox, 1voy, 1voz, 1vp0)			
Macromolecular chains	480 (split over 4fy1, 4fy2, 4fy3, 4fy4, 4fy5, 4fy6, 4fy7, 4fy8, 4fy9, 4fya)			
No. of chemical components in PDB dictionary	15218			
Entry with the highest number of ligands (not including water)				
Total ligand molecules	5357 (split over 3i8f, 3i8g, 3i8h, 3i8i)			
Unique ligands	19 (3arc)			

**(b):** EMDB holdings on 12/12/2012.

	No. of released volume maps, all methods	Single particle and icosahedral only
Total	1560	1245
Better than 5 resolution	37	32
Systems of more than 10MDa	129‡	123

† Excluding entries that have been declared by the depositors to have been solved by X-ray (or NMR) in combination with other methods, which are counted as ‘hybrid’.

‡ Often only the molecular weight of the repeating unit is reported, so this count is likely to be underestimated.

**Table 2:** Areas of collaboration between the wwPDB partners

Area of collaboration	Examples
Policy issues	Definition of mandatory items and data for deposition, *e.g.* structure-factor amplitudes
Archive releases	Weekly updates exchanged between all sites; simultaneous release of identical copies of the archive
Validation standards	Validation task forces provide recommendations that the wwPDB partners implement
Format specifications	PDBx/mmCIF, PDBML, PDB
Chemical component descriptions	Detailed description of new chemical components, including ideal coordinates
Deposition and annotation procedures	Agreement on common procedures to describe, for example, quaternary structure or Ramachandran outliers, or the use of reference resources, *e.g.* for sequence annotation
Archive quality and remediation	Regular review of quality, consistency and integrity issues leading to large-scale archive-wide remediation
Journal interactions	Recommendations with respect to wording of deposition and release requirements; coordination of publications and data release

**Table 3:** Roles of structural bioinformatics resources and organizations such as PDBe, wwPDB and EMDataBank

Areas of activity	PDBe	wwPDB	EMDataBank
Community interactions	Involvement in CCP4 (Winn *et al.*, 2011 ▶), CCPN (Vranken *et al.*, 2005 ▶) and CCP-EM	Validation task forces for X-ray crystallography and NMR (Berman *et al.*, 2010 ▶)	Validation task force for EM (Henderson *et al.*, 2012 ▶)
	Advisory committee	Consultations with IUCr	Operation of http://emdatabank.org portal
		Interactions with journals	Organization of consultative workshops
		Advisory committee	Advisory committee
Community challenges	Involvement in CAPRI (Janin Wodak, 2007 ▶), CASD-NMR (Rosato *et al.*, 2012 ▶)	Involvement in CASP (Moult *et al.*, 2011 ▶), CASD-NMR (Rosato *et al.*, 2012 ▶)	Organization of EM Modelling challenge (Ludtke *et al.*, 2012 ▶)
Changes and support of file formats	Interactions with and support for CCPN (Vranken *et al.*, 2005 ▶)	Specification of PDB, PDBx/mmCIF, PDBML formats (Westbrook *et al.*, 2005 ▶)	Specification of formats for EM volume maps, Fourier shell-correlation curves and map segmentations
Data models and ontologies	Involvement in crystallization ontology (Newman *et al.*, 2012 ▶), CCPN (Vranken *et al.*, 2005 ▶)	PDBx	EMDB data model, mapping to PDBx
Support for new methods for structure determination		Task forces for small-angle scattering (SAS) and hybrid methods	
Deposition, annotation, validation, archiving and distribution of data		Management and distribution of PDB and BMRB archives	Management and distribution of EMDB archive
Integration with other resources	SIFTS (Velankar *et al.*, 2005 ▶, 2013 ▶) mappings between PDB proteins and many other resources	Cross-links to reference resources, *e.g.* UniProt (UniProt Consortium, 2012 ▶) for protein sequences	Cross-links to reference resources
Advanced services exposing structural information	*PDBePISA*, *PDBeMotif*, *PDBeFold* and many more (Velankar *et al.*, 2012 ▶)		

**Table 4:** Analysis of cryo-EM/ET-related publications in selected journals for the year 2011 and corresponding data depositions in the EMDB A PubMed (http://www.ncbi.nlm.nih.gov/pubmed/) search was carried out for each journal using the query: ‘cryoelectron microscopy[MeSH Terms] OR ((Models, Molecular[MeSH Terms] OR Models, Structural[MeSH Terms]) AND ‘electron microscopy’)’. The results were then scrutinized manually for relevant hits.

	Tomography			Single particle		
Journal	Publications	Publications with depositions	Fraction (%)	Publications	Publications with depositions	Fraction (%)
*Cell*						
*J. Mol. Biol.*	3	1	33	10	6	60
*Mol. Cell*				2	1	50
*Proc. Natl Acad. Sci. USA*	7	3	43	24	12	50
*EMBO J.*	1	0	0	7	5	71
*J. Struct. Biol.*	9	1	11	6	2	33
*Nature Struct. Mol. Biol.*				9	6	67
*Science*				2	2	100
*J. Biol. Chem.*	1	1	100	11	3	27
*J. Virol.*	5	2	40	10	3	30
*Nature (London)*				7	3	43
*Structure*	2	0	0	13	6	46
Total	28	8	29	101	49	49
